# Protein C concentrations in severe sepsis: an early directional change in plasma levels predicts outcome

**DOI:** 10.1186/cc4946

**Published:** 2006-06-15

**Authors:** Andrew F Shorr, Gordon R Bernard, Jean-Francois Dhainaut, James R Russell, William L Macias, David R Nelson, David P Sundin

**Affiliations:** 1 Department of Medicine, Section of Pulmonary and Critical Care Medicine, Washington Hospital Center, Washington, DC, USA; 2 Department of Medicine, Division of Allergy, Pulmonary and Critical Care Medicine, Vanderbilt University, Nashville, Tennessee, USA; 3 Department of Intensive Care and Emergency Medicine, Cochin-Port Royal University Hospital, Paris 5 René Descartes University, Paris, France; 4 Critical Care Research, St Paul's Hospital and University of British Columbia McDonald Research Laboratories, Vancouver, Canada; 5 Lilly Research Laboratories, Eli Lilly and Co., Indianapolis, Indiana, USA

## Abstract

**Introduction:**

Protein C, because of its central role in hemostasis, plays an integral role in the host response to infection. Protein C depletion, resulting from increased consumption, degradation, and/or decreased synthesis, is characteristic of sepsis and has been shown to predict morbidity and mortality. The objective of this study was to determine whether early directional changes in protein C levels correlate with outcome.

**Methods:**

Patients in the Recombinant Human Activated Protein C Worldwide Evaluation in Severe Sepsis (PROWESS) clinical trial were assessed and categorized by baseline protein C (*n *= 1574). Deficiency was categorized as: severe deficiency, protein C levels ≤ 40% of normal protein C activity (*n *= 615, 39% of patients); deficient, protein C levels 41–80% of normal protein C activity (*n *= 764, 48.5% of patients); and normal, >80% of normal protein C activity (*n *= 195, 12.4% of patients). Logistic regression analysis of 28-day mortality for placebo patients was used to investigate whether baseline and day 1 protein C levels were independent risk factors for mortality. The impact of treatment with drotrecogin alfa (activated) (DrotAA) was also assessed.

**Results:**

Protein C levels at baseline and day 1 were independent risk factors in placebo patients. If baseline protein C levels of severely deficient placebo patients remained ≤ 40% at day 1 their odds of death increased (odds ratio = 2.75, *P *< 0.0001), while if levels improved to >40% by day 1 their risk of death decreased (odds ratio = 0.43, *P *= 0.03). If baseline protein C levels of placebo patients were >40% but decreased by ≥ 10% on day 1, their risk of death increased (odds ratio = 1.87, *P *= 0.02). DrotAA treatment improved protein C levels by day 1 compared with placebo (*P *= 0.008) and reduced the risk of death in severely deficient (≤ 40%) patients at baseline. Treatment also decreased the number of severely protein C deficient (= 40%) patients and decreased the number of deficient (41–80%) patients and normal (>80%) patients who had a ≥ 10% decrease in protein C levels by day 1.

**Conclusion:**

Baseline protein C levels were an independent predictor of sepsis outcome. Day 1 changes in protein C, regardless of baseline levels, were also predictive of outcome. The association of DrotAA treatment, increased protein C levels, and improved survival may partially explain the mechanism of action.

## Introduction

The protein C pathway, because of its central role in hemostasis, plays an integral role in the host response to infection. Activated protein C inactivates coagulation factors, enhances fibrinolysis, and at high concentrations reduces the release of inflammatory cytokines [1-6]. Due to increased consumption, degradation, and/or decreased synthesis, protein C deficiency is characteristic of severe sepsis – with the onset of protein C deficiency probably occurring before clinical diagnosis of organ dysfunction [7-9]. Numerous studies have demonstrated that decreased circulating levels of protein C in septic patients are associated with increased morbidity and mortality [7-11]. The extent of protein C deficiency, assessed at the time of diagnosis, correlates with increased morbidity and mortality, but only as a threshold [12]; that is, only severe protein C deficiency (protein C levels ≤ 40% of normal protein C activity) correlates with decreased survival. Continued protein C deficiency or the development of protein C deficiency within approximately one day of diagnosis, however, has been correlated with early death [12].

Drotrecogin alfa (activated) (DrotAA) has been shown to improve survival in patients with severe sepsis [13] and to increase protein C levels [14]. This effect appears to be unique to protein C, as similar findings were not observed with protein S and antithrombin III. The treatment effect appeared independent of baseline protein C measurements [15], although *a priori *it was hypothesized that protein-C-deficient patients would derive the most benefit from treatment.

We hypothesized that early (baseline to day 1) directional changes in protein C (naturally occurring or from DrotAA treatment) would correlate with outcome. Since lower protein C levels appear to correlate with worse outcomes, we explored whether the observed change from baseline in the first day (either an increase or decrease) would contribute meaningful additional information to baseline levels of protein C, with respect to predicting outcome. In addition, we explored whether treatment with DrotAA would reduce the number of patients with day 1 decreases in protein C levels or increase the number of patients that improved from severe protein C deficiency.

## Materials and methods

### Patients

The Recombinant Human Activated Protein C Worldwide Evaluation in Severe Sepsis (PROWESS) trial was conducted in accordance with ethical principles that have their origin in the Declaration of Helsinki and are consistent with good clinical practices and applicable laws and regulations. The trial design, patient disposition, inclusion/exclusion criteria, and results of the pivotal PROWESS clinical trial have been described in detail previously [13]. Briefly, PROWESS was a multicountry (164 sites in 11 countries), randomized, placebo-controlled clinical trial of DrotAA (Xigris^®^; Eli Lilly and Company, Indianapolis, IN, USA) in adult patients with severe sepsis. All investigative sites obtained approval for the study from their institutional review boards. Written informed consent was obtained from all patients or their legal representatives. Although protein C activity levels were measured in the PROWESS trial, patients with missing baseline protein C activity values were excluded from these analyses.

### Samples

In the PROWESS trial, plasma samples were obtained at baseline and daily through study day 7. A central laboratory (Covance Central Laboratory Services, Indianapolis, IN, USA) performed all assays. The protein C activity assay was performed on a STA^® ^coagulation analyzer using the STA^®^-Staclot^® ^Protein C kit (Diagnostica Stago, Asnieres-Sur-Seine, France), which has a coefficient of variation of 7.5%. Protein S measurements were performed on the STA^® ^coagulation analyzer using the STA^®^-Staclot^® ^Protein S kit (Diagnostica Stago). The antithrombin III activity was quantitated using a chromogenic activity assay (Stachrome ATIII; Diagnostica Stago). IL-6 antigen levels were measured by enzyme immunoassay (Quantikine Human IL-6 HS kit; R&D Systems, Minneapolis, MN, USA).

### Statistical methods

The relationship between baseline protein C levels and clinical variables was assessed with Spearman rank correlation when both variables were continuous, and was assessed with the Wilcoxon rank-sum test (for groups with two levels, for example comorbidities) when continuous protein C levels were compared between two classes.

Protein C classes were defined prospectively [13,15] into normal (>80% of normal protein C activity), deficient (41–80% of normal protein C activity), and severely deficient (≤ 40% of normal protein C activity). The protein C status was evaluated to determine whether it was a significant risk factor for mortality among PROWESS placebo patients. Multivariable logistic regression was used to adjust for six risk factors (Acute Physiology and Chronic Health Evaluation (APACHE) II score, age, baseline IL-6 level, presence of comorbidities, presence of any dependencies as determined by ability to conduct activities of daily living [16], and urosepsis) previously found to be significant predictors of outcome in analyses of data from PROWESS placebo patients [15]. Baseline protein C classes were initially included. Variables assessing protein C change from baseline were subsequently included in stepwise logistic regression. In some cases the moderately deficient protein C and normal protein C classes were combined for analysis of mortality by baseline protein C activity levels, after it was determined that there was no increased risk in the odds of death (odds ratio = 0.89, *P *= 0.07, comparing 41–80% of normal protein C activity with >80% of normal protein C activity; see Table 2), over the time frame analyzed.

For patients with both baseline and day 1 protein C measurements, treatment differences of antithrombin, IL-6, and protein S levels at baseline and day 1, and their changes, were compared with Wilcoxon rank-sum tests. Survival patterns were illustrated with Kaplan-Meier estimates and were compared using log-rank tests. Statistical analyses were performed using SAS version 8.02 software (SAS Institute Inc., Cary, NC, USA).

## Results

The PROWESS trial enrolled 1690 patients, of which 850 received DrotAA and 840 received placebo. Baseline protein C measurements were obtained for 1574 patients (799 receiving DrotAA and 775 receiving placebo). Patients who had missing baseline protein C values did not significantly differ from the overall population in baseline characteristics, disease severity measures, or outcomes (data not shown). Values ranged from 5% to 200% of normal, with an average of 50.6 ± 26.7% (mean ± standard deviation). Using prospectively defined criteria, patients were classified as either severely deficient (≤ 40% activity, *n *= 615, 39.1% of patients), deficient (41–80% activity, *n *= 764, 48.5% of patients), or normal (>80% activity, *n *= 195, 12.4% of patients). It should be emphasized that the levels of protein C reported relate to levels of endogenous inactivated protein C. In addition, the reported protein C values do not reflect intravenously administered DrotAA.

### Relationship between baseline protein C class, clinical and demographic characteristics, and 28-day mortality

Table 1 presents seven baseline characteristics of PROWESS patients as they relate to baseline protein C class and treatment group. Six of the baseline characteristics (APACHE II score, age, log IL-6, presence of comorbidities, presence of dependencies, and urosepsis) have previously been shown to predict outcome among PROWESS placebo patients [15] and were specifically chosen to present for that reason (see Table 2). Significant correlations were observed across baseline protein C classes for the APACHE II score and IL-6 (*P *< 0.0001 for both). Comorbidities, dependencies, and septic shock also had a significant association with baseline protein C levels (all *P *≤ 0.007). The 28-day mortality among the PROWESS placebo patients was significantly higher in the severely deficient (≤ 40% activity) protein C group than in the deficient (41–80% activity, *P *< 0.0001) and normal (>80% activity, *P *= 0.006) protein C groups. The deficient and normal groups, however, did not differ significantly from each other (*P *= 0.71).

### Baseline and day 1 changes in protein C levels predict mortality in PROWESS placebo patients

To determine whether low or decreasing protein C places patients at a high risk of mortality, analyses adjusting for six previously defined significant risk factors (APACHE II score, age, log IL-6, presence of comorbidities, presence of dependencies, and urosepsis) [15] were performed on data from PROWESS placebo patients (Table 2). Baseline severe protein C deficiency (≤ 40% activity) was associated with a significantly higher risk of death (adjusted odds ratio = 1.75, *P *= 0.003) than those patients without severe deficiency (baseline protein C level 41–80% and >80% activity).

Changes in protein C activity level in the first day also significantly predicted the risk of death (Table 2). If placebo patients were severely deficient at baseline and remained severely deficient on day 1, their odds of death were 2.74 times higher than other placebo patients (*P *< 0.0001). Placebo patients with deficient (41–80%) and normal (>80%) protein C activity levels at baseline (for example, baseline protein C >40% in Table 2) who had a ≥ 10% decrease in protein C levels on day 1 also had a significantly increased risk of death (odds ratio = 1.87, *P *= 0.02), compared with patients who did not have a decrease of this magnitude. If placebo patients were severely deficient (≤ 40%) at baseline but improved to >40% activity by day 1, their risk of death was significantly reduced compared with patients whose protein C activity levels remained ≤ 40% (odds ratio = 0.43, *P *= 0.03). Other variables associated with change did not enter the model. For instance, no significant increased risk was observed for day 1 protein C decreases in the severely deficient subgroup (≤ 40% activity). In contrast, no significant decreased risks were observed for day 1 protein C increases in the deficient (41–80% activity) and normal (>80% activity) subgroups (for example, baseline levels >40% activity).

**Figure 1:**
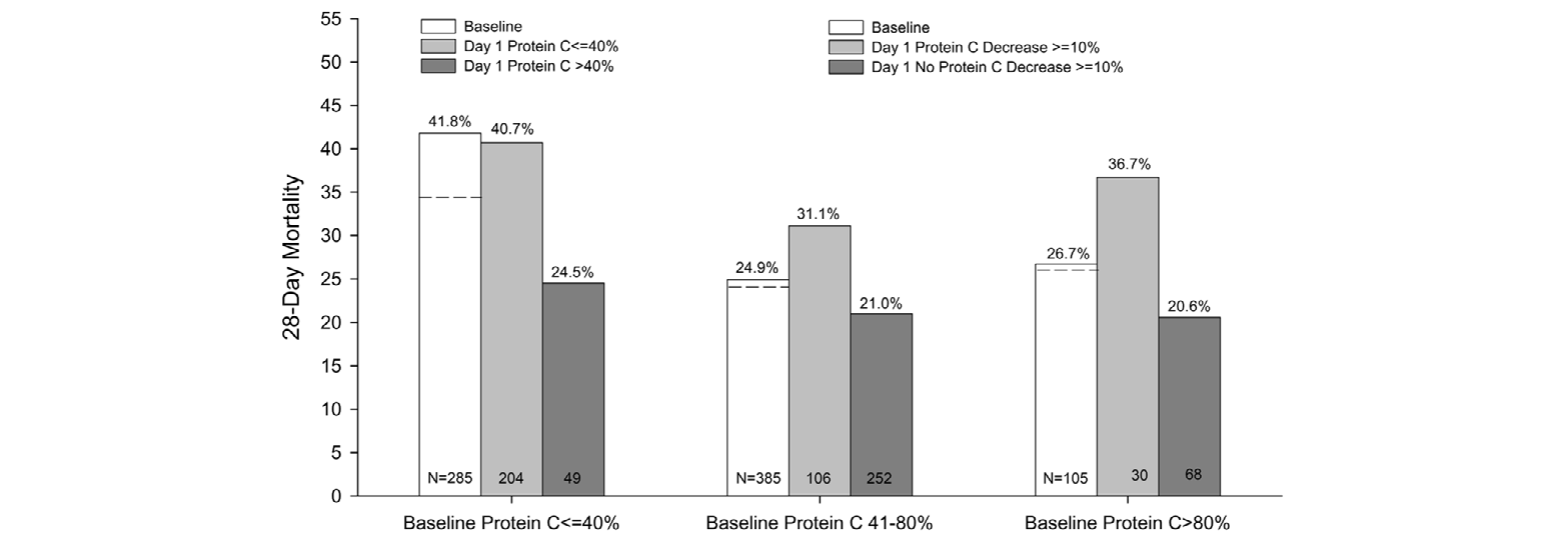
PROWESS 28-day placebo mortality rates: effect of baseline protein C class. Placebo patient baseline and day 1 protein C levels were assessed and patient outcomes at 28 days determined. Twenty eight-day mortalities, based on baseline protein C and day 1 protein C levels are presented. Mortality of severely deficient patients at baseline increased if protein C levels remained ≤ 40% and decreased if protein C levels increased to >40%. Mortality of deficient (41–80%) and normal (>80%) patients increased if there was a ≥ 10% drop in protein C levels and decreased if there was no drop ≥ 10%. The area/% above the dashed lines in all baseline bar graphs represent patients that died before a day 1 protein C measurement was performed.

To illustrate the significance observed in day 1 protein C changes, the PROWESS placebo mortality rates, based on baseline levels and first day protein C changes, are presented in Figure 1. Of the severely deficient placebo patients (Figure 1, left-hand bar graphs), 7.4% did not survive to day 1 for a second protein C measure (area above the dotted line). After removing patients who died before the day 1 protein C measurement was taken (dotted line), the mortality of severely deficient patients at baseline (34.9%) increased to 40.7% if their protein C levels remained ≤ 40% and decreased to 24.5% if their levels rose above 40%.

In the middle set of bar graphs in Figure 1, 1.0% of moderately deficient placebo patients died before a second measure could be taken (area above dotted line). After removing patients who died before a day 1 protein C measurement was taken (dotted line), the mortality of deficient patients at baseline (24.0%) increased to 31.1% if there was ≥ 10% decrease in their protein C levels and decreased to 21.0% if no decrease ≥ 10% occurred.

Finally, in the right-hand set of bar graphs in Figure 1, 1.0% of placebo patients with normal protein C levels died before a second measure could be taken (area above dotted line). After removing patients who died before a day 1 protein C measurement was taken (dotted line), the mortality of patients with normal protein C levels at baseline (26.0%) increased if there was a decrease in their protein C levels ≥ 10% (36.7%) and decreased if there was no drop in their protein C levels ≥ 10% (20.6%).

### Day 1 improvement of protein C levels: effect of DrotAA

Although randomization in the PROWESS trial created a placebo group with slightly higher median baseline protein C levels (*P *= 0.06, Table 3), by day 1, DrotAA-treated patients had significantly increased protein C levels (*P *= 0.008). The median day 1 change in protein C showed a 6% increase for DrotAA-treated patients, compared with a 0% change for placebo (*P *< 0.0001).

Table 4 demonstrates the specificity of the DrotAA effect. There was no significant difference between treatment groups in day 1 levels or the day 1 change in two other markers of coagulation, protein S (*P *= 0.41 and *P *= 0.59, respectively) and antithrombin III (*P *= 0.61 and *P *= 0.88, respectively). Although there was no significant difference between treatment groups in the day 1 levels of the inflammation marker IL-6 (*P *= 0.44), the day 1 change in IL-6 was significantly reduced in the DrotAA group (*P *= 0.006). There was a slight imbalance of higher IL-6 levels in the DrotAA group at baseline (*P *= 0.08).

The proportion of PROWESS patients in each baseline protein C category that improved or worsened by day 1 with DrotAA treatment is illustrated in the bottom half of Table 3. DrotAA significantly increased the proportion of severely deficient patients whose protein C levels improved to deficient or normal levels (that is to say >40% activity, *P *< 0.0001). In addition, DrotAA significantly decreased the proportion of deficient patients who had a ≥ 10% drop in protein C (*P *= 0.0002) and numerically reduced the proportion of normal patients who had a ≥ 10% drop in protein C (*P *= 0.09).

**Figure 2:**
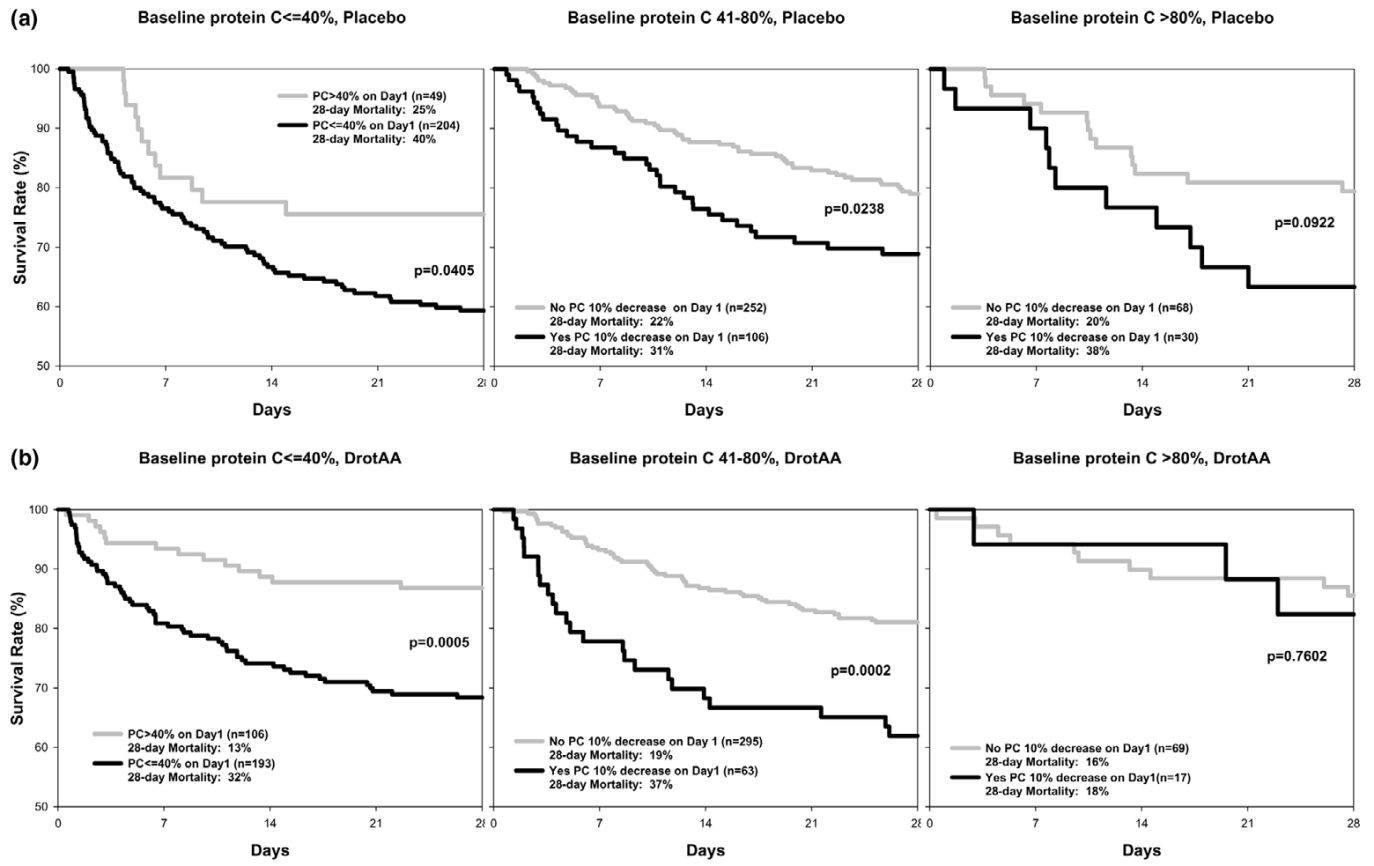
PROWESS 28-day survival curves based on baseline and day 1 protein C activity levels. Baseline and day 1 protein C levels for patients by treatment group were assessed and 28-day outcomes determined. Kaplan-Meier survival curves of each group are presented. Curves were compared using the log-rank test and *P *< 0.05 was considered significant. Severely deficient (≤ 40%), moderately deficient (41–80%) and normal (>80%) baseline protein C categories are presented from left to right. **(a) **Survival curves of placebo patients. In all cases, if day 1 protein C levels improved (to >40%) or remained stable (no ≥ 10% decrease), survival was higher than if protein C levels remained ≤ 40% or decreased by ≥ 10%. The improvement was significant only for severely deficient and deficient patients.**(b) **Survival curves of PROWESS drotrecogin alfa (activated) (DrotAA) patients. A similar pattern was observed in DrotAA-treated patients as in placebo patients. In all cases, 28-day survival was higher if day 1 protein C levels improved (to >40%) or remained stable than if they remained ≤ 40% or decreased by ≥ 10%. The improvement was significant only for severely deficient and deficient patients.

Survival curves (Kaplan-Meier estimates) of PROWESS placebo and DrotAA-treated patients, based on baseline protein C class and the day 1 change in protein C levels, are presented in Figure 2. Curves of placebo patients (Figure 2a) who were severely deficient (≤ 40%) and deficient (41–80%) at baseline were significantly better in patients whose day 1 protein C levels improved to >40% or stabilized (no decrease ≥ 10%) than those whose day 1 protein C values remained ≤ 40% or had a ≥ 10% decrease. In the relatively small subgroup of patients with normal (>80%) baseline protein C, the same trend was observed. In general, this same pattern was observed in DrotAA-treated patients (Figure 2b), although the degree of increase in mortality of patients with normal (>80%) baseline protein C and a ≥ 10% decrease was not observed.

## Discussion

This analysis demonstrates that the directional change of protein C levels correlates with outcome and the change from baseline in the first day provides more information on the eventual prognosis than do baseline protein C levels alone in individuals with severe sepsis. Additionally, the risk for death associated with various protein C levels seems to follow a threshold effect with clear risk classes. Furthermore, early changes in protein C levels, in combination with baseline protein C levels, predict outcome. Patients whose protein C levels fail to stabilize (deficient patients and normal patients) or fail to improve (severely deficient patients) faced a higher risk of death. Finally, DrotAA appears to alter survival through its direct impact on endogenous protein C levels.

The current study differs from and builds on a previous study investigating the interaction of protein C levels and DrotAA treatment [15]. For that past assessment, all protein-C-deficient patients were pooled into a common group and no effort was made to separate the moderately and severely deficient protein C classes. In the present analysis, risk for mortality was not continuous within the deficient group. The likelihood of death was very high in severely deficient protein C patients (protein C levels ≤ 40% of normal), while the risk of death in patients with deficient (41–80% of normal) and normal (>80% of normal) protein C levels was equivalent. It is possible, however, that the risk of death in moderately deficient and normal protein C groups would not be the same if protein C was analyzed over a greater period of time.

Our observation that mortality increased if baseline protein C levels were >40% and if a patient's day 1 protein C levels fell by ≥ 10% is novel. These results are consistent with previous studies that suggested decreases in protein C levels precede overt clinical symptoms [7-9] and may be predictive of increased mortality [7-11]. Hence, future investigations should focus on measuring protein C levels as soon as possible after sepsis is suspected and then evaluate the role for serial protein C measurements. This could potentially provide a more rapid and accurate assessment of the patient's status. If such studies confirm that specific rapid declines in protein C levels can be readily detected, and further that they precede clinical deterioration, this information could be used to guide therapy.

Results from this study also suggested that improvements in outcome hinge on increases in protein C levels over the first day following diagnosis and baseline protein C measurement. These improvements were observed to occur in patients not treated with DrotAA and could be a component of the natural host response or a result of the numerous currently available clinical interventions such as infection source control, antibiotics or other measures. On the other hand, the results presented here provide supportive evidence that DrotAA treatment specifically increases endogenous protein C levels.

Regardless of the reason for improvement, the change from baseline data to day 1 data emphasized that it is important for these changes to occur rapidly. If protein C levels decrease by as little as 10% on day 1, mortality increases significantly among most individuals. Moreover, DrotAA treatment significantly reduces mortality in the severely deficient protein C group, probably reflecting these patients being more likely to have increased protein C levels at day 1 because of treatment with DrotAA. Conversely, DrotAA-treated patients with moderately deficient (41–80% of normal) or normal (80% of normal) protein C levels at baseline were less likely to have a ≥ 10% drop in protein C levels. This fact probably explains that the treatment effect of DrotAA is less robust in these populations. In lower risk patients DrotAA prevented the progression of low-risk individuals to high-risk status, presumably by stabilizing protein C levels. This apparent association between DrotAA treatment, increased protein C levels, and improved survival may suggest that the mechanism of action for DrotAA is primarily reflective of its direct impact on protein C levels.

The relationship between protein C and DrotAA appears unique. For example, the increase in protein C levels with DrotAA treatment was not observed in a different anticoagulant (antithrombin III), although derangements in other coagulation markers have been previously observed to improve with DrotAA treatment [13,14]. The significant reduction from baseline levels of IL-6 could be from the anti-inflammatory activity of protein C, which stems from its antithrombotic activity or from a yet to be described mechanism. The potential insight gained by incorporation of dynamic assessments of protein C reinforces the plausibility of why DrotAA is efficacious in severe sepsis. Additional prospective studies looking at more rapid diagnosis of sepsis, early and serial assessment of individual changes in protein C levels, titration of DrotAA dose, and duration of DrotAA treatment using serial protein C assessment are clearly needed to further clarify the results presented here.

There are important limitations to the present study. As a result of the exclusion criteria (patients at high risk of bleeding, patients with low platelet count, and so on), the actual prevalence of patients with low protein C levels in severe sepsis may be higher than observed in this study. Likewise, although many of the parameters assessed in this study were prospectively defined, most of the analyses in this study were performed in a retrospective manner. Finally, limitations in the availability of samples (patients with missing baseline protein C values) prevented a more robust analysis of the early daily changes in protein C.

## Conclusion

In summary, the current study confirmed that baseline protein C levels are an independent predictor of outcome in severe sepsis patients. Early changes in protein C levels (such as day 1) were also significant risk factors in combination with baseline protein C levels. The risk associated with protein C levels appears to be categorical rather than continuous in nature. The data imply that DrotAA treatment decreases mortality in two ways: by raising protein C levels above 40% of the normal threshold, and by reducing the number of moderately deficient patients and normal patients who had a decrease in their baseline protein C levels ≥ 10%. Finally, an association between DrotAA treatment, increased protein C levels, and improved survival exists that suggests a mechanism of action.

## Key messages

• 	A decline in protein C levels in patients with severe sepsis and septic shock identifies a population at high risk for death.

• 	Dynamic, temporal analysis of changes in protein C levels provides more insight into probable outcomes than a static, one-time assessment.

• 	The risk for death associated with various protein C levels seems to follow a threshold effect with clear risk classes.

• 	Drotrecogin alfa (activated) appears to exert its effect on mortality reduction in part through increasing levels of protein C.

## Abbreviations

APACHE = Acute Physiology and Chronic Health Evaluation; DrotAA = drotrecogin alfa (activated); IL = interleukin; PROWESS = Recombinant Human Activated Protein C Worldwide Evaluation in Severe Sepsis.

## Competing interests

Eli Lilly and Company provided support for this study. GRB, J-FD, JRR, and AFS have all participated in Eli Lilly and Company-sponsored clinical trials, and have all served as consultants for Eli Lilly and Company. WLM, DRN, and DP are employees and stockholders of Eli Lilly and Company.

## Authors' contributions

WLM, DRN, GRB, DPS, and AFS participated in the conception and design of the study. GRB, J-FD, and JRR participated in the PROWESS clinical trial and contributed to data collection. All authors contributed to development and conduct of the principal analyses and participated in drafting the manuscript. All authors contributed to revision of the manuscript. All authors read and approved the final manuscript.

## Figures and Tables

**Table 1:** Baseline characteristics and 28-day mortality of PROWESS patients by baseline protein C level and treatment group

Parameter	Baseline protein C ≤ 40% of normal		Baseline protein C 41–80% of normal		Baseline protein C >80% of normal	
	
	Placebo (*n *= 285)	DrotAA (*n *= 330)	Placebo (*n *= 385)	DrotAA (*n *= 379)	Placebo (*n *= 105)	DrotAA (*n *= 90)
APACHE II score*	26.1 ± 8.1	25.6 ± 7.7	24.3 ± 7.8	23.9 ± 7.5	23.9 ± 7.3	23.8 ± 6.7
Age (years)	61.4 ± 16.9	60.9 ± 17.1	60.0 ± 16.7	59.9 ± 17.8	61.9 ± 14.6	61.4 ± 16.4
Log IL-6*	7.4 ± 2.1	7.5 ± 2.2	6.1 ± 2.0	6.2 ± 2.0	4.7 ± 1.8	4.7 ± 1.7
Urosepsis (%)	9.8	10.3	12.2	8.7	3.8	14.4
Comorbidites^a ^(%)**	16.5	15.5	20.0	19.8	35.2	34.4
Dependencies^b ^(%)**	25.3	21.8	29.6	29.3	38.1	33.3
Septic shock (%)**	76.8	80.6	72.2	66.0	59.0	55.6
28-day mortality (%)	41.8	27.6	24.9	24.0	26.7	15.6

DrotAA, drotrecogin alfa (activated). Patients were prospectively categorized on the basis of their baseline protein C activity levels (normal, >80%; deficient, 41–80% of normal; and severely deficient, ≤ 40% of normal). ^a^Any chronic health points from the (APACHE) II classification system. ^b^Patient considered dependent if they were dependent in one or more activities on the Activities of Daily Living scale [16]. *Significantly different between protein C classes (*P *< 0.05) using Spearman rank-correlation with baseline protein C levels. **Significant (*P *< 0.05) association with baseline protein C levels using Wilcoxon rank-sum tests comparing the "yes" versus "no" classifications

**Table 2:** Protein C and additional measures as risk factors in PROWESS placebo patients

Parameter	Baseline protein C			Baseline and day 1 protein C		
	
	Odds ratio^a^	95% confidence interval	*P *value	Odds ratio^a^	95% confidence interval	*P *value
APACHE II score^b^	1.31	1.16–1.48	<0.0001	1.33	1.17–1.51	<0.0001
Age^c^	1.17	1.10–1.24	<0.0001	1.17	1.10–1.24	<0.0001
Log IL-6^d^	1.48	1.22–1.80	<0.0001	1.30	1.06–1.60	0.010
Comorbidity	2.00	1.32–3.03	0.001	1.80	1.17–2.78	0.008
Dependencies^e^	1.70	1.17–2.46	0.006	1.81	1.23–2.68	0.003
Urosepsis	0.47	0.25–0.87	0.020	0.42	0.22–0.79	0.008
Baseline protein C ≤ 40%	1.75	1.21–2.53	0.003	2.74	1.75–4.30	<0.0001
Baseline protein C ≤ 40% and day 1 protein C >40%	-	-	-	0.43	0.20–0.93	0.030
Baseline protein C >40% and day 1 protein C 10% decrease	-	-	-	1.87	1.12–3.11	0.020

APACHE, Acute Physiology and Chronic Health Evaluation. All patients with baseline protein C values from the PROWESS trial (*n *= 1574) were included in analyses. Risk factors used were previously found to be significant predictors of outcome in analyses of data from PROWESS [15]. ^a^Based on multivariable logistic regression; values >1 indicate increased risk, values <1 indicate lower risk. ^b^Per 5-point increase in baseline score. ^c^Per 5-year increase in age. ^d^Per 10 times increase in baseline IL-6. ^e^Patient was considered dependent if they were dependent in one or more activities on the Activities of Daily Living scale [16].

**Table 3:** Baseline and Day 1 protein C activity: effect of drotrecogin alfa (activated) (DrotAA) and proportion of patients that improved or worsened by baseline protein C class

Parameter	Treatment group		
	
	Placebo (*n *= 709)	DrotAA (*n *= 743)	*P *value
Protein C activity^a^			
Baseline	50% (34, 68)	47% (31, 64)	0.06
Day 1	47% (30, 72)	52% (36, 72)	0.008
Day 1 change	0% (-10, 9)	6% (-4, 16)	<0.0001
Proportion of patients improved or worsened (% of patients)^b^			
Baseline protein C ≤ 40% and day 1 protein C >40%	19.4	35.5	<0.0001
Baseline protein C 40–80% and day 1 protein C decreased ≥ 10%	29.6	17.6	0.0002
Baseline protein C >80% and day 1 protein C decreased ≥ 10%	30.6	19.8	0.09

**[AU Query: Delete any previous text that is a repeat of information in main body of article] **^a^Summary statistics reported as the median (25th percentile, 75th percentile), and *P *values from Wilcoxon rank-sum tests (*P *< 0.05 significant). ^b^Improved, baseline protein C ≤ 40% and day 1 protein C >40%; Worsened, baseline protein C 41–80% or >80% and a day 1 decrease in protein C ≥ 10%.

**Table 4:** Baseline and day 1 values for protein S, antithrombin III, and IL-6 by treatment group in the PROWESS trial

Parameter	Treatment **g**roup		
	
	Placebo (*n *= 709)	Drotrecogin alfa (activated) (*n *= 743)	*P *value
Protein S activity			
Baseline	39% (24, 59)	35% (22, 57)	0.09
Day 1	36% (23, 57)	35% (22, 56)	0.41
Day 1 change	0% (-9, 7)	0% (-10, 9)	0.59
Antithrombin III			
Baseline	60% (45, 75)	59% (44, 75)	0.50
Day 1	59% (45, 75)	59% (44, 76)	0.61
Day 1 change	0% (-10, 9)	0% (-8, 7)	0.88
IL-6			
Baseline	436 (126, 2338)	523 (156, 2701)	0.08
Day 1	199 (68, 717)	211 (77, 685)	0.44
Day 1 change	-157 (-1073, -6)	-225 (-1922, -27)	0.006

Summary statistics reported as the median (25th percentile, 75th percentile), and *P *values from Wilcoxon rank-sum tests (*P *< 0.05 significant).
